# ASO Author Reflections: Novel 2D Histopathological Markers for the Risk Stratification of Sentinel Node Metastasis in the Era of Adjuvant Systemic Therapy for Primary Cutaneous Melanoma

**DOI:** 10.1245/s10434-025-17610-4

**Published:** 2025-06-19

**Authors:** Sophie E. Orme, Marc D. Moncrieff, Gerald Saldanha

**Affiliations:** 1 Norfolk & Norwich University Hospitals NHS Trust, Norwich, UK; 2 Norwich Medical School, University of East Anglia, Norwich, UK; 3 University Hospitals of Leicester NHS Trust, Leicester, UK

## Past

With the advent of adjuvant systemic therapy for resected primary cutaneous melanoma, sentinel node biopsy (SNB) has become a gateway to access such therapies by identifying micrometastatic stage III disease.^1,2^ Breslow thickness (BT), a one-dimensional measure, remains the principal staging criterion in the *American Joint Committee on Cancer Staging Manual,* 8th edition.^3^ Although BT correlates well with prognosis, AJCC stage alone has proven inadequate in stratifying patients by their risk of sentinel node positivity.^3^ This limitation has prompted renewed interest in identifying novel primary tumor biomarkers to better predict sentinel node (SN) status and survival outcomes. Saldanha et al. previously proposed a two-dimensional metric, the calculated tumor area (CTA), which demonstrated independent prognostic value and potential superiority over BT.^4^ By offering a more granular assessment of invasive tumor burden, two-dimensional markers may better reflect metastatic potential, particularly to the sentinel node, and thereby improve prognostic precision.

## Present

The latest analysis by Orme et al. corroborates the findings of Saldanha et al., demonstrating a statistically significant difference in disease-specific survival between patients stratified into high- and low-risk groups by using a statistically modelled optimal cut point for CTA.^5^ However, in contrast to earlier work, CTA was not found to be an independent predictor of sentinel node status. The authors attributed this to variability introduced by the subjective estimation of tumor proportion. In the present study, we describe for the first time a purely objective quantitative metric, the Simplified Breslow Area (SBA), derived from the transformation of tumor thickness and invasive width, where SBA equals the sum of the natural logarithm of Breslow thickness and the natural logarithm of invasive width. Multivariate regression analysis identified SBA as the strongest independent predictor of sentinel node status; it outperformed lymphovascular invasion, ulceration, and Breslow thickness.

## Future

If these findings are validated in larger international cohorts, they would suggest that two-dimensional markers, such as SBA, have greater predictive value for both sentinel node status and survival than the current model founded on BT. The clinical application of SBA still requires further definition. As digital pathology becomes more widely adopted and as the potential of artificial intelligence in this domain continues to grow, the objective, multidimensional assessment of tumor characteristics may become more reproducible and routinely achievable; in such a scenario, 2D markers, such as SBA, may supersede BT as more accurate representations of microscopic tumor burden in future classification systems.

Alternatively, SBA may be used as an adjunct in prognostication and clinical decision-making for specific patient groups, such as those with pT1b-pT2a tumors, in whom the AJCC classification alone cannot reliably stratify nodal risk. This may involve incorporation into composite risk models that combine SBA with other clinicopathologic features known to have predictive value. In the future, patient selection for SNB may be further refined by the integration of histopathological factors, such as SBA, with molecular prognostic data obtained through gene expression profiling or immunohistochemistry; however, these techniques require further study before their widespread clinical application.
